# The social and cultural shaping of cybersecurity capacity building: a comparative study of nations and regions

**DOI:** 10.1007/s00779-021-01569-6

**Published:** 2021-05-10

**Authors:** Sadie Creese, William H. Dutton, Patricia Esteve-González

**Affiliations:** Global Cyber Security Capacity Centre, Department of Computer Science, University of Oxford, Oxford, UK

**Keywords:** Cybersecurity,, Culture,, Society,, Internet,, Region,, Development

## Abstract

This paper presents an empirical study of the social and cultural aspects of cybersecurity capacity building in 78 nations. While nations within geographically defined regions might be expected to share similar attitudes, values, and practices around cybersecurity, this analysis finds that regional differences can be explained largely by cross-national differences in development and the scale of Internet use. These results question the centrality of regions in shaping social and cultural attributes directly tied to cybersecurity capacity. However, the analysis identifies some countries with greater and some with lesser levels of maturity in capacity building than expected only on the basis of their development and scale of Internet use. Further research focused on the dynamics of under- and over-performance of different nations might illuminate where regional contexts could place a brake on, or provide an impetus for, under- or over-performance in cybersecurity capacity building. That said, national development and the scale of Internet use are the most explanatory of cultural attitudes, values, and practices of societies tied to cybersecurity, such as trust on the Internet.

## Introduction

Cybersecurity is about ‘the technologies, processes, and policies that help to prevent and/or reduce the negative impact of events in cyberspace that can happen as the result of deliberate actions against information technology by a hostile or malevolent actor’ [7]. Given the challenges of preventing such negative impacts, increasing attention has been focused on building the capacity of nations to be resilient in the face of security problems, including the development of social and cultural aspects of capacity building. While early research on computer and Internet security focused on the technical aspects of securing systems, the increasing centrality and diffusion of the Internet has added new dimensions to capacity building, including a range of attitudes, values, and practices of Internet users that define a socio-cultural or social and cultural dimension of cybersecurity capacity building.

As the percentage of the world’s population using the Internet for more significant activities has grown, so has the interest in building a secure cyber space. The capacity of a nation to build online security might well depend on the attitudes, values, and practices of Internet users, such as their awareness of security risks, their online habits and practices, and the prioritisation they place on their security. Moreover, there are social and political factors out of the users’ direct control that might affect their cybersecurity, such as their perceptions of the rights they have in relation to the protection of their personal information online, and the structures in place to permit users to report threats to their privacy or security. Some of these determinants are designed and implemented at the national or supranational level, such as by the European Union (EU) and the Organization for American States (OAS). Some examples are the new EU Cybersecurity Strategy adopted in December 2020 and the symposia and programmes around cybersecurity that the OAS develops in the region.^1^

Therefore, cybersecurity capacity building might depend in part on the attitudes, values, and practices of individual Internet users, some of which might well be tied to national and regional policies, structures, and traditions, such as in data protection or freedom of expression. We have assumed that many of these individual level attitudes, values, and practices are associated with one another to form a coherent dimension of cybersecurity capacity building. This paper describes these social and cultural aspects tied to cybersecurity and examines whether they are quite unique or related closely enough to define a single dimension of cybersecurity capacity building. Given this analysis, we move to a description of the maturity of capacity building on this dimension across nations and regions, based on scales informed by international consultation.

Are differences in the social and cultural aspects of capacity building accounted for by their regional contexts? We began this analysis with the expectation that geography would make a significant difference and help explain social and cultural patterns of variation across nations. This paper addresses this question through a cross-national and cross-regional comparative analysis of capacity building in 78 nations, finding that regional differences are largely explained by two key national variations in the scale of Internet use and the nation’s development.

The paper uses original data from the application of the ‘Cybersecurity Capacity Maturity Model for Nations’ (CMM). The CMM is an analytical framework that allows cross-nationally comparable reviews of national cybersecurity capacity. The framework is based on rating levels of maturity across five interrelated but separable dimensions of capacity building, including a dimension on the cyber-related risks in society which is called ‘Cybersecurity Culture and Society’. Key social and cultural underpinnings of enhancing cybersecurity capacity are more specifically identified and differentiated from factors tied to other critical dimensions of capacity building.

Quantitative analysis of our 78-nation data set in combination with relevant secondary data is then used to address how nations and regions differ on the social and cultural dimension of capacity building and why. Region is often viewed as a surrogate of cultural and societal differences. The cultural makeup of a nation might well be shaped by regional cultures and in turn shape social and cultural approaches to cybersecurity. While regions reflect many broad social and cultural differences, they might also be tied to specific attitudes, values, and practices directly related to cybersecurity, and also associated with levels of development. We therefore use multivariate analyses to consider whether region or other factors, such as wealth and the centrality and scale of Internet use, explain the variations in social and cultural aspects of cybersecurity capacity building.

Our analyses suggested that regional differences in social and cultural underpinnings of cybersecurity exist, but that these differences can be accounted for by national economic and socio-political variables related to the level of development and scale of national Internet use. This led the analysis to identify three countries underestimated by our model and two overestimated, for which we argued that possible explanations for these over- and under-performing cases might entail regional considerations.

The paper begins by outlining alternative expectations around the relationships between socio-cultural aspects of cyber security capacity building, then describes our data, the analytical strategy, and findings. We conclude by discussing the implications and limitations of this study along with suggestions for further research.

## Theoretical expectations and related research

The central focus of research on computer security and, more recently, cybersecurity has been on technology—technical advances in security. However, there has been a developing tradition of work on the social shaping of the Internet and related information and communication technologies (ICTs) that informs this study [6, 11, 31]. This literature is distinctly different from research on national cultures, such as Hofstede’s cultural dimensions theory [24]. Instead, the social shaping of technology (SST) focuses on social or cultural factors directly tied to shaping the design, implementation, use and implications of technology. But also, within the mainstream computer science community, major overviews of cybersecurity have recognized that ‘technology is only one aspect of security, and is arguably not even the most important’ ([7], p. 75). This same review identifies ‘nontechnological factors’ to include economics, psychology, education, convenience and ease of use, law, and organizational factors ([7], p. 75-86).

Some observers have found low-income countries to have been globally innovative in the area of digital media, such as in the early use of electronic payment systems. From this perspective, Internet users in these nations might be leapfrogging more traditional Internet users, suggesting that they can respond rapidly when new technology is relatively advantageous. In these countries, social and cultural factors might be an impetus for innovation [34]. Others have found negative cultural responses to the Internet, such as a distrust of information online, to be more prominent among those who have not used or experienced the technology [15]. The significance of the Internet as an experience technology offers one rationale for expecting possible less positive attitudes toward the Internet in low-income nations given their slower pace of Internet diffusion. Similarly, some research supports the centrality of education to instilling a cybersecurity culture, which would also disadvantage low-income nations [17]. Other evidence, such as global surveys, have suggested that the most dominant values and attitudes of Internet users are relatively common across the world [14]. If so, social and cultural differences might not be key to understanding any relative advance or lag in capacity building in low- and medium-income nations.

Despite a tradition of research on SST, there is not a strong body of research on the social shaping of cybersecurity and what we define as the social and cultural aspects of cybersecurity [26]. There has been work on the idea of a cybersecurity mindset [12], which is related to capacity building, and literature on information cultures (see, for example, [33]). The present study is most closely related to cross-national studies of the use of the Internet across various sectors of society. The studies in the literature of cybersecurity analyse national indicators of overall cybersecurity capacity, without analysing individually the social and cultural dimensions potentially included in such indicators (see, for example, [5, 8, 9]). In this area, major studies include work on a Cyber Readiness Index designed to ‘evaluate a country’s maturity and commitment to cybersecurity’ ([37], p. 4; [10]). [32] have used international comparative approaches to identify factors shaping cyber readiness. The Belfer Center for Science and International Affairs at the Harvard Kennedy School has focused primarily on the US [21]. The National Institute of Standards and Technology (NIST) has developed a NIST Cyber Security Framework [2]. The Cybersecurity Capacity Maturity Model for Nations (CMM) is one of a number of maturity frameworks, but the framework employed in this study is one of the few that seek to incorporate what might be called the social and cultural dimension of cybersecurity. For a comparison of the existing frameworks, see [3, 35].

The literature and our analyses of factors shaping the overall cybersecurity capacity of a country has demonstrated the importance of countries’ wealth and size, along with the scale of Internet use in explaining the variability of maturity in cybersecurity [8, 9, 13]. Moreover, as there is a growing theme around the abilities of democratic institutions to be as agile in implementing security policy and practice, such as in response to protests [38], we incorporated in our analysis variables related to the character of each society that have been tied to security policy and practice, broadly captured as its democratic and governmental context.

Regional initiatives could make a difference in shaping the beliefs and values associated with cybersecurity [1]. How important are the regions to the development of cybersecurity? Are there unique issues within regions, or are regional organizations simply closer geographically and culturally for communication about key developments in policy, technology, and practice?

Drawing from original field research and secondary data, this paper contributes to the literature on cybersecurity by developing indicators of the social and cultural aspects of cybersecurity across 78 nations and their regions to determine whether particular regions do leap frog or lag behind other regions [22, 27]. We hypothesized that the cultural and social aspects of relevance to capacity building are shaped by regional differences, even when controlling by cross-national differences in the scale and the economic and governmental development of nations.

## Field research and data

This study addresses this hypothesis on the basis of cross-sectional data drawn from cybersecurity capacity reviews conducted in 78 nations. All 78 were studied, using the ‘Cybersecurity Capacity Maturity Model for Nations’ (CMM) of the Global Cyber Security Capacity Centre [18]. The CMM is a framework to assess the maturity of a country regarding its cybersecurity capacity across five dimensions, including the dimension on cybersecurity culture and society: (1) Cybersecurity Policy and Strategy; (2) Cybersecurity Culture and Society; (3) Cybersecurity Education, Training and Skills; (4) Legal and Regulatory Frameworks; and (5) Standards, Organisations, and Technologies.

The culture and society—or social and cultural—dimension of capacity building is broad, but tied specifically to aspects of the values, attitudes, and practices of Internet users that have the potential to directly shape the safe use of the Internet by individuals, households, government, civil society, and business and industry. This dimension is itself split into five different ‘factors’, which include a cybersecurity mindset, trust, and confidence in use of the Internet, user understanding of personal information protection online, awareness of ways to report issues of cybersecurity or privacy, and the degree that media educate the public about cybersecurity issues.

In turn, each factor includes multiple ‘aspects’ which were calibrated by a set of direct empirical indicators of cybersecurity capacity rated on five stages of maturity: (1) Start-Up, (2) Formative, (3) Established, (4) Strategic, and (5) Dynamic. Although the maturity stage of each aspect is characterized by different indicators, the meaning of each maturity stage has a common definition across aspects allowing for their comparison. Therefore, within the dataset, each aspect is considered an ordinal variable that can take a value between 1 and 5 according to the increasing maturity on each scale defined in the CMM [18].

### Data collection

The CMM review process involved a two to four-person review team from Oxford (or a partner institution)^2^ traveling to each country for three to four days and conducting about ten modified-focus group sessions in the field with key representatives of national stakeholder clusters, enumerated in Box 1. Participants in the discussions were identified prior to the field visit and clustered into groups based on their expertise in each dimension of the CMM. Each of the ten sessions ran for about 2 hours and had between 5 to 15 participants—all identified as stakeholders in the nation’s cybersecurity, but representing different institutions and different kinds of expertise.

**Box 1:** Stakeholder clusters participating in the modified-focus groups

Academia, Civil Society groups, and Internet Governance Criminal Justice and Law Enforcement Cyber Task Force Cybersecurity Incident Response Teams (CSIRT) Defence and Intelligence Community Government Ministries Information Technology Leaders from Government and the Private Sector International Partners Legislators and other Policy Owners, such as Appointed Experts Private Sector and Business Representatives of Critical National Infrastructures	

Two different approaches of the GCSCC to data collection were used to gauge the maturity stage of all aspects in the CMM. The primary research approach involved qualitative coding using mixed methods [29, 30, 39]. The methods included desk research prior to the field work, followed by modified-focus groups and interviews in the field to generate ordinal scores for each dimension of the CMM. The team used ‘modified-focus groups’ rather than traditional focus group facilitation in order to gain a multi-stakeholder perspective on the current status of all aspects of the model, rather than to generate a wide range of ideas. Each session focused on one or two dimensions of the model to ensure that each dimension was discussed in-depth and by more than one modified-focus group.

During each session, the review team asked questions to guide discussions around indicators of relevance to the dimensions being considered by that group of stakeholders. Each session was recorded with the consent of all participants, assuring the participants that the recordings would be used solely for the purpose of writing the review, and accurately representing their views.^3^ This meant for example that no one would be quoted without their express permission. However, the resulting ratings of maturity could be used as data in our research.

Prior to the field research, desk research was conducted to ensure that the moderators and research team were aware of basic information about the nation, including governance, business and industry, and cybersecurity operations and officers in the country. After the field work, the evidence provided in different sessions was triangulated with a separate desk research phase, sometimes requiring further documents or interviews to fill any gaps.^4^

The second approach involved GCSCC collaboration with the Organization of American States (OAS) and the Inter-American Development Bank (IDB) to develop an online tool to be completed by OAS member states. Based on the CMM and adapted to the particular context of Latin America and the Caribbean countries, the online tool was available in English and Spanish. OAS sent the survey with a secure access code to the online tool to their member states through official communication channels. They asked their national point of contact in each country to distribute the survey to relevant national experts with the most relevant knowledge to ensure the provision of the most reliable information about cybersecurity in the country. Multiple respondents in each country participated. The OAS technical team aggregated responses and undertook desk research to supplement the data required to arrive at maturity scores for each dimension. The aggregated scores were then sent to each member state for validation, leading to the final maturity stages of each aspect, which were published in [25].

The research team at GCSCC subsequently reviewed the data from these two different approaches (field and online data collection) to make sure they could be combined and to identify any outliers. The overall sample had 47 countries reviewed once by the GCSCC during the period 2015–2020, and 31 countries surveyed by OAS.

The research team analysed data at the aspect level, since this was the finest level of detailed data collected through both methods. During the course of validating the data, only one nation was identified as having extreme scores that raised doubts. In that case, we did further analyses and decided that the outlying scores on some maturity levels could not be validated by additional interviews and documentation and the country was therefore removed from the data set.

Generally, there was remarkable coherence, based on such indicators as inter-item reliability, and construct validity, across the nations that participated in the study. Throughout the analysis phase, the team continued to look for anomalies that might be attributed to the different methodological approaches but found clear and reliable patterns indicating that the data from the two methods could be validly combined.

### Countering the risk of an ethnocentric perspective

In developing indicators of capacity and gauging maturity across nations, there is a risk of imposing ethnocentric criteria of evaluation. This is a risk to all comparative research, but of particular concern in cybersecurity since it has been led by the most economically developed nations that could invest early in ICTs. However, the project was designed to avoid an ethnocentric approach through multiple strategies. First, the research team has been international from the outset, including researchers from Asia, Latin America and the Caribbean, North America, Europe, Africa, and Oceania. Secondly, the model and the development of each of its aspects has been informed by international expert consultation and collaboration through an ongoing process and two reviews of the CMM itself. The establishment of the GCSCC and the development of the CMM led to collaboration with strategic partners such as the OAS, the World Bank, CTO, and ITU to conduct CMM reviews in their member states and parter countries. Overtime, this also led to the establishment of a constellation of centres, including the Oceania Cyber Security Centre (OCSC), Melbourne, and the Cybersecurity Capacity Centre for Southern Africa (C3SA), Cape Town, both of which lead the deployment of the CMM in their respective regions. In the future, the GCSCC plans to establish further partnerships in both Asia and Latin America. Thirdly, a national CMM review takes place on invitation of the respective government, which is involved in the process from the beginning, owns the outcoming CMM report, and decides on its publication. Fourthly, as discussed in our methodological approach, the GCSCC review process involves multiple stakeholders in each nation to review all judgements of maturity levels in order to reach a mutual agreement on their validity. This not only prevents the team from misinterpreting observations or comments during the review but also ensuring that more local knowledge is embedded in the review. While adding months to the research, this process increases the internal validity of the assessment and diminishes risks of ethnocentric biases.

### Indicators of social and cultural maturity

Given original data collected in the field by the Oxford research team and its collaborating partners, the analysis is anchored in an unusually rich set of data that is focused on the social and cultural aspects of cybersecurity—not surrogate indicators—and gathered systematically to maximise the reliability of the team’s approach. Table 1 describes the factors and aspects related to this dimension of the CMM Cybersecurity Culture and Society, and summarizes the indicators of capacities that gauge the maturity stage of a country on each aspect. This results in nine variables on cultural and social aspects of cybersecurity that can take values between 1 and 5, indicating an increasing level of maturity under the CMM.

**Table 1:** Factors, aspects, and indicators of the CMM dimension Cybersecurity Culture and Society

Factor	Aspect	Summary of indicators
Cybersecurity Mindset	Government	Knowledge and priority of cybersecurity in the practices, habits, and strategic planning within and across government agencies.
	Private Sector	Knowledge and priority of cybersecurity in the practices, habits, routine, and strategic planning across business and industries.
	Users	Knowledge and priority of cybersecurity in the practices, habits, and strategic planning of Internet users and the overall population.
Trust and Confidence on the Internet	Internet Services	Provision and development of Internet infrastructures across all services; how operators of Internet provide secure online services; users’ control over personal data.
	E-Government Services	Provision of e-services offered by the government, their security measures and protection of personal information; feedback and impact assessments.
	E-Commerce Services	Provision of e-commerce services; reliability of the payment systems and personal information policies; accessible and comprehensible terms and conditions; feedback and impact assessments on privacy.
User Understanding of Personal Information Protection Online	User Understanding of Personal Information Protection Online	Knowledge of personal information protection online across users and stakeholders within the public and private sectors; practices and mechanisms to ensure privacy and security.
Reporting Mechanisms	Reporting Mechanisms	Channels in place for reporting online incidents, their coordination, their promotion, and their effectiveness.
Media and Social Media	Media and Social Media	Coverage of cybersecurity subjects by the traditional media and social media.

Over time, the CMM has included new aspects to adapt the model to the changing nature of cybersecurity capacity, including three new aspects added to the social and cultural dimension (2.5 User Understanding of Personal Information Protection, 2.6 Reporting Mechanisms, and 2.7 Media and Social Media). This study considers the most recently deployed version of the CMM, as 71 countries were reviewed under this framework. We also included in the sample seven additional countries reviewed under a previous CMM framework. Although this results in missing data on three of the new aspects for these seven countries, we still have the data on the six other aspects included in this dimension, enabling us to gain a relatively reliable maturity score for this dimension across all the countries.

Multivariate approaches were used to determine whether the aspect scores within each factor of each cybersecurity dimension were sufficiently correlated to ensure that they could be reliably combined in a single average for each factor. These analyses helped to confirm that they were measuring the same underlying trait, allowing us to build a single variable for the maturity of the social and cultural dimension, as a weighted average of all the aspects within this CMM dimension. This is the dependent variable of our study, that we call the Social and Cultural Dimension (SCD). Table 2 displays the descriptive statistics of this variable jointly with its comprised aspects and factors for the countries in the sample.^5^

**Table 2:** Descriptive statistics of the Social and Cultural Dimension (SCD) and its components. Standard deviations are in parentheses next to the corresponding mean. If a factor has a unique aspect, we provide only the data at the factor level to avoid duplication of information

Dimension / Factor / Aspect	Obs.	Mean (S.Dev.)	Min	Max
Social and Cultural Dimension (SCD)	78	1.73 (0.48)	1.00	3.00
2.1 Cybersecurity Mind-set	78	1.76 (0.50)	1.00	3.00
2.1.1 Government	78	1.85 (0.59)	1.00	4.00
2.1.2 Private Sector	78	1.94 (0.58)	1.00	3.00
2.1.3 Users	78	1.50 (0.57)	1.00	3.00
2.2 Confidence and Trust on the Internet	78	1.78 (0.63)	1.00	3.50
2.2.1 Internet Services	78	1.63 (0.64)	1.00	3.00
2.2.2 E-government	78	1.80 (0.66)	1.00	3.50
2.2.3 E-commerce	78	1.90 (0.77)	1.00	4.50
2.3 User Understanding of Personal Information Protection Online	71	1.47 (0.60)	1.00	3.00
2.4 Reporting Mechanisms	71	1.76 (0.62)	1.00	3.00
2.5 Media and Social Media	71	1.87 (0.58)	1.00	3.50

The most general observation to note from Table 2 is the low average values of aspects and factors. This demonstrates that countries in the sample are, on average, in an early phase of cybersecurity capacity building on the social and cultural aspects of cybersecurity. They all fell at maturity stages between Start-Up (value 1) and Formative (value 2). For example, factor 2.3 User Understanding of Personal Information Protection Online is rated on average as the least mature across all the nations, with a value slightly below 1.50. However, this value is not far from the highest value 1.94, corresponding to the sample countries’ average maturity stage of the cybersecurity mindset in the private sector (aspect 2.1.2 Private Sector).

Nevertheless, the range of these cybersecurity variables show sufficient variability for meaningful analyses, given maximum observations above the Formative stage (value 2), and a few countries with stages above the Strategic level (value 4). For example, aspect 2.2.3 E-Commerce has one country (Switzerland) with a maturity stage of 4.50, which is between the Strategic and Dynamic stage. Our study relies on explaining the variability of the data through the different regions of the sample countries, controlling by other factors that may shape SCD, as explained below.

### Indicators of factors shaping social and cultural maturity levels

Based on previous studies, we considered relevant determinants of national overall cybersecurity capacity when the CMM assessment was conducted. In particular, the approximate wealth of a country, as indicated by its GDP per capita in 2010 dollars, and its total population size. We estimated the centrality of the Internet in a country by its percentage of Internet users, which is drawn from secondary data. We estimated the total number of Internet users as an indicator of scale.^6^ Three of the variables in these analyses (GDP per capita, total population, and number of Internet users) have a different scale to the rest of the variables and a highly skewed distribution given our country sample, which includes a sizeable proportion of low-income nations. To address these issues, we applied the natural logarithm of the value of each of these three variables.

We approximated the national democratic context by the number of years that each country has been a democracy on the basis of criteria defined by [4]. Their definition of democracy entails fulfilling three conditions:^7^ (1) ‘the executive is directly or indirectly elected in popular elections and is responsible either directly to voters or to a legislature’; (2) ‘the legislature (or the executive if elected directly) is chosen in free and fair elections’; (3) ‘a majority of adult men has the right to vote’.

Additional features that help to describe the governmental context of a country include the level of corruption in the country and the efficiency of the government in implementing its policies. Control of corruption could be a useful indicator of administrative structures geared to reducing weak institutional practices, such as lax security. Regulatory quality could be a useful indicator of the ability to implement regulations, one aspect of building cybersecurity capacity. We approximate these two features by including two variables defined by [28].^8^ The first variable, control of corruption, captures the perception that public power is not exercised for private gain and interests. The second variable, regulatory quality, captures perceptions of the ability of each respective government to formulate and implement policies and regulations. Table 3 displays the descriptive statistics of these seven secondary contextual variables.

**Table 3:** Descriptive statistics of secondary contextual variables. Standard deviations are in parentheses next to the corresponding mean

Variable	Obs.	Mean (S. Dev.)	Min	Max
Total population, log	78	15.23 (2.14)	10.87	19.37
GDP per capita (2015 US$), log	78	8.39 (1.10)	6.13	11.28
Number of Internet users, log	78	14.37 (2.12)	9.75	18.82
Percentage of Internet users	78	50.01 (24.05)	4.71	98.26
Years of democracy	78	28.85 (29.14)	0.00	172
Control of corruption	78	−0.12 (0.76)	−1.51	1.98
Regulatory quality	78	−0.12 (0.69)	−2.36	1.85

We can observe in Table 4 that some of these variables are strongly and positively intercorrelated. In particular, there is a strong correlation between those variables related either to the scale of the country (total population and number of Internet users) or to its level of what we call development (GDP per capita, percentage of Internet users, years of democracy, control of corruption, and regulatory quality). For example, nations with higher GDP per capita are more likely to have a higher percentage of Internet users (centrality), more years of democratic governance, more control of corruption, and higher quality of regulation.

**Table 4:** Relationships among contextual variables: correlation matrix with Pearson’s correlation coefficients. Symbols *, **, *** indicate levels of significance 0.05, 0.01, 0.001, correspondingly

	T. pop.	GDP pc	N. users	P. users	Y. dem.	C. corr.	R. qual.
Total population	1.00						
GDP per capita	−0.26*	1.00					
Number users	0.95***	−0.00	1.00				
Percentage users	−0.17	0.87***	0.12	1.00			
Years democracy	−0.10	0.64***	0.02	0.51***	1.00		
Control corruption	−0.48***	0.63***	−0.36**	0.47***	0.60***	1.00	
Regulatory quality	−0.14	0.67***	0.02	0.60***	0.58***	0.68***	1.00

Given the number of variables of interest, some of which are highly correlated, there is a risk of misinterpreting the findings of multivariate analyses. For example, the correlation coefficient between the number of Internet users and total population is 0.95. If total population is entered in a regression, it could explain the variance that might be attributed to number of Internet users. Instead of implying that number of users was not significant, it might be more valid to consider both indicators as measuring the same underlying variable, in this case ‘scale’. By using factor analyses, we were able to reduce the number of variables in our regression to a smaller set of factors and thereby reduce these problems of collinearity between some variables. In addition, as our sample of countries has a limited number of observations (78), the analysis gains degrees of freedom for our estimations by reducing the number of variables.

Using factor analyses, we confirmed that seven independent variables could be well represented by two factors. Table 5 displays the orthogonal solution where the two first factors account for the largest proportion of variance, and the corresponding factor loadings for the two retained factors, representing the correlation between the seven variables and each factor. The number of Internet users and total population could be combined to represent a ‘scale’ index (Factor 2), and the percentage of Internet users, GDP per capita, years of democracy, control of corruption, and regulatory quality could be combined to reflect a ‘development’ index (Factor 1). The factors are not completely independent statistically, but by rotating the factors the same two factor pattern is sharply defined (Table 6). This factor analysis generated two new variables, a Development and Scale index, with descriptive statistics detailed in Table 7.

**Table 5:** Factor analysis/correlation (unrotated) and factor loadings. LR test independent vs. saturated: Chi2(21)=628.33 Prob>Chi2=0.00

Factor	Eigenvalue	Difference	Proportion	Cumulative
Factor 1	3.39	1.46	0.61	0.61
Factor 2	1.94	1.54	0.35	0.96
Factor 3	0.40	0.37	0.07	1.03
Factor 4	0.03	0.04	0.00	1.03
Factor 5	−0.01	0.05	−0.00	1.03
Factor 6	−0.06	0.06	−0.01	1.02
Factor 7	−0.11	.	−0.02	1.00
Variable		Factor 1	Factor 2	Uniqueness
Total Population		−0.49	0.85	0.03
GDP per capita		0.88	0.24	0.16
Number users		−0.27	0.96	0.01
Percentage users		0.80	0.34	0.25
Years democracy		0.68	0.22	0.49
Control corruption		0.81	−0.14	0.33
Regulatory quality		0.74	0.22	0.40

**Table 6:** Rotated factor loadings. Orthogonal varimax rotation

Variable	Factor 1	Factor 2	Uniqueness
Total population	−0.17	0.97	0.03
GDP per capita	0.91	−0.07	0.16
Number users	0.07	0.99	0.01
Percentage users	0.86	0.05	0.25
Years democracy	0.71	−0.02	0.49
Control corruption	0.71	−0.41	0.33
Regulatory quality	0.77	−0.05	0.40

**Table 7:** Descriptive statistics of Development and Scale variables. Standard deviations are in parentheses next to  the corresponding mean

Variable	Obs.	Mean (S. Dev.)	Min	Max
Development	78	0.00 (0.97)	−1.88	3.03
Scale	78	0.00 (1.00)	−2.06	2.08

Finally, to approximate the regional variable, we used the single categorical variable Region that clusters countries according to their continent; our sample has 20 observations in Africa, 10 in Asia, 10 in Europe, 31 in Latin America and the Caribbean (Americas in short, not including Canada or the USA), and 7 in Oceania.

## Methodology and analysis of the comparative data

Our initial analytical strategy was to determine if regions helped to explain the differences in the maturity of cultural and societal aspects of cybersecurity for our sample countries. We used complementary methodologies to explain such differences.

First, we analysed our dependent variable, the Social and Cultural Dimension (SCD), with descriptive statistics per region, describing the average maturity stage of the sample countries in each region. Then, we used a *t*-test to validate if any identified difference between the average of two regions was statistically significant. In a *t*-test, the null hypothesis is that the average of two different groups is the same. Therefore, we used a *t*-test to accept or reject the null hypothesis that the average maturity stage of SCD for each pair of regions was the same. We complemented the *t*-test results with box plots that allowed to us compare the distribution of the sample data within and across the different regions. Finally, we ran a linear regression with the 78 countries in our sample where the dependent variable was the SCD, and the independent variables were Region, Development, and Scale.

### Regional differences

**Fig. 1:**
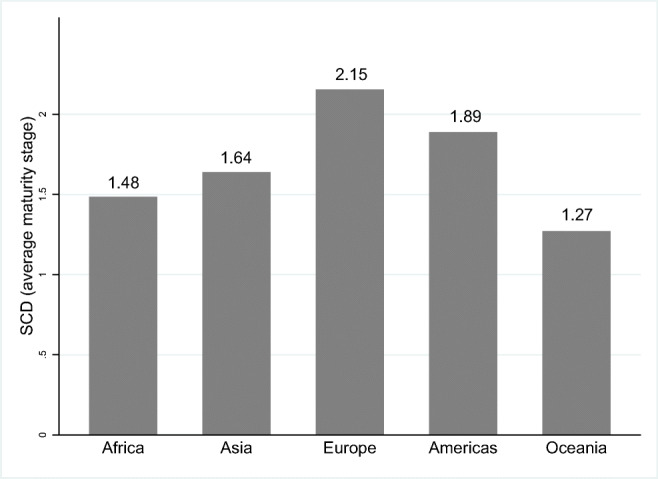
Average maturity stage of the Social and Cultural Dimension (SCD) in each region

Without controlling for development and scale, we observe some zero-order differences across regions (Fig. 1). The most obvious is the relatively greater maturity in SCD of Europe compared to all other regions, and the relatively low level of cybersecurity culture and society indicators in Oceania (Fig. 1).^9^ That said, it is critical to remember that these are not random or complete samples of nations in each region, and that our countries over-represent lower-income nations in most regions.

Table 8 displays the results of the *t*-test, examining whether the difference between the average maturity stage of the Social and Cultural Dimension for the two different regions is statistically significant. The table shows that Europe and the Americas have a significantly larger average maturity stage than the other regions, and that the difference of averages between Europe and Americas regions observed in Fig. 1 is not large enough to reject the null hypotheses that such averages are the same.

**Table 8:** *T*-test comparing the average maturity stage of the Social and Cultural Dimension (SCD) between regions. Symbols ***, **, *, + indicate, correspondingly, levels of significance <0.001, <0.01, <0.05, and <0.1

Pair of regions	Combined obs.	*T*-test coefficient
Africa–Asia	30	−1.10
Africa–Europe	30	−4.12**
Africa–Americas	51	−3.71***
Africa–Oceania	27	1.73
Asia–Europe	20	−2.67*
Asia–Americas	41	−1.66
Asia–Oceania	17	2.28*
Europe–Americas	41	1.55
Europe–Oceania	17	4.88***
Americas–Oceania	38	4.59***

On the other extreme, the region Oceania has a significantly smaller average maturity than the other regions. The average maturity stage of the Social and Cultural Dimension is not statistically significant between the African and Asian regions, leading us to conclude that the averages of these two groups are approximately the same.

These main results must be taken with care as we observe some weak significance levels between two pairs of regions. Concretely, the null hypothesis that Africa and Oceania regions have the same average maturity stage is accepted, and the null hypothesis that Asia and Americas regions have the same average maturity stage is accepted as well.

**Fig. 2:**
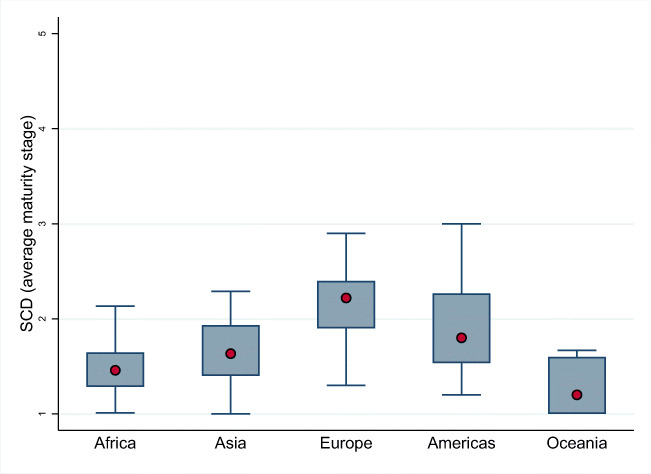
Box plots with the distribution of the average maturity stage of the Social and Cultural Dimension (SCD) in each region

Figure 2 illustrates the distribution of maturity on the Social and Cultural Dimension for the observations in each regional group, where the median position is signalled with a red point and the values inside the box correspond to the values in the interquartile range (percentiles 25th to 75th). The box plot of Europe is clearly above the boxes of the rest of regions, without any overlapping except with the box of the Americas region, and with a median value (2.22) above the rest of regions. The box plots of the Americas and Asia regions overlap significantly. Although these two regions have a relatively similar median value (1.80 for the Americas and 1.63 for Asia), the distributions look quite different with a larger variance in the Americas region than in the Asia region. This could be driven by the smaller number of observations in the Asia region (10) compared to the number of observations in the Americas group (31). The box of Oceania is clearly in a lower position than the rest of regions, overlapping with the bottom of the boxes of Africa, Asia, and Americas, although this overlap is more significant with the box of Africa. However, the median value of the Oceania distribution (1.20) is lower than the other regional distributions (1.46 for Africa, 1.63 for Asia, and 1.80 for the Americas), and the distribution of the Oceania region has a higher proportion of observations with the minimum maturity level compared to the rest of regions. When looking at the distribution of Africa and Asia regions, there are some slight differences in the variability of the values in each subsample and the median values but, overall, these two regional distributions look quite similar, and the two boxes overlap significatively. In essence, the distribution plots in Fig. 2 confirm the results of the tests in Table 8.

### Multivariate analysis

Knowing that region appears to matter when viewed on its own, the analysis moved to a more multivariate perspective. We employed a simple linear regression with only Region and our Development and Scale variables to explain the variability in national levels of maturity of the SCD. The regressions in Table 9 use Europe as the base category as this region has the maximum median value. Similarly, as with the previous analysis, the regression in column (1) estimates that countries in the sample belonging to a different region than Europe have a lower average maturity stage except for those countries in the Americas region. This penalisation can downgrade the average maturity stage for SCD slightly above 0.5 units for the Asian region, around 0.7 units for the African region, and almost 0.9 units for the Oceania region.

**Table 9:** Regression to estimate on the Social and Cultural Dimension (SCD). Robust standard errors in parenthesis below the corresponding coefficient. Europe is the base category for region; symbols +, *, **, *** indicate levels of significance 0.1, 0.05, 0.01, 0.001, correspondingly

	(1)	(2)	(3)	(4)	(5)
Development		0.29***		0.30***	0.34***
		(0.05)		(0.04)	(0.04)
Scale			0.19***	0.20***	0.18***
			(0.05)	(0.04)	(0.04)
Africa	−0.67***	−0.15	−0.69***	−0.15	
	(0.16)	(0.15)	(0.15)	(0.14)	
Asia	−0.52**	−0.14	−0.60**	−0.21	
	(0.19)	(0.16)	(0.19)	(0.15)	
Americas	−0.27	−0.01	−0.25	0.01	
	(0.17)	(0.15)	(0.16)	(0.13)	
Oceania	−0.88***	−0.39*	−0.62***	−0.10	
	(0.18)	(0.17)	(0.18)	(0.17)	
Constant	2.15***	1.83***	2.14***	1.80***	1.73***
	(0.15)	(0.12)	(0.14)	(0.12)	(0.03)
N	78	78	78	78	78
*R*^2^	0.30	0.51	0.42	0.64	0.61

However, when the analysis includes the variables Development and Scale in the regression, the detected differences across regions become insignificant. Also, the analysis shows that this change is statistically driven mainly by the Development variable. Moreover, adding these two new variables allows the model to increase the explanatory power of our model for estimating the average maturity stage of SCD.

Specifically, the *R*-squared increases from 0.30 in estimation (1) when only region variables are included to 0.64 in estimation (4) when all the variables are included. This last value is very similar to the *R*-squared value 0.61 of estimation (5) when only the variables Development and Scale are included, entirely dropping the regional categories. Overall, it is evident that the differences of the average social and cultural maturity ratings across these regions in Fig. 1 are based on the differences in the development and scale levels of such countries, rather than their regional context. Therefore, in contrast to our initial expectation, the findings do not support the hypothesis that regions play a significant role in shaping the social and cultural aspects of cybersecurity.

### Countries over- and under-performing

Our finding was that the variables Development and Scale are the most powerful explanatory variables for Social and Cultural Dimension (SCD) of cybersecurity capacity, muting the significance of the regional effects. We built on that insight to determine what countries were over- or under-performing. That is, what countries had a maturity level significantly higher or lower than predicted on the basis of Development and Scale. To accomplish this, we calculated the estimated average maturity stage for SCD for each country in our sample (${\hat{y}}_i$) according to the coefficients in the regression number (5) in Table 9. Therefore, we used Eq. (1) to calculate the estimated average maturity stage for our country sample.

1 $$ {\hat{y}}_i=1.73+0.34\mathrm{Development}+0.18\mathrm{Scale}\kern1em $$

**Fig. 3:**
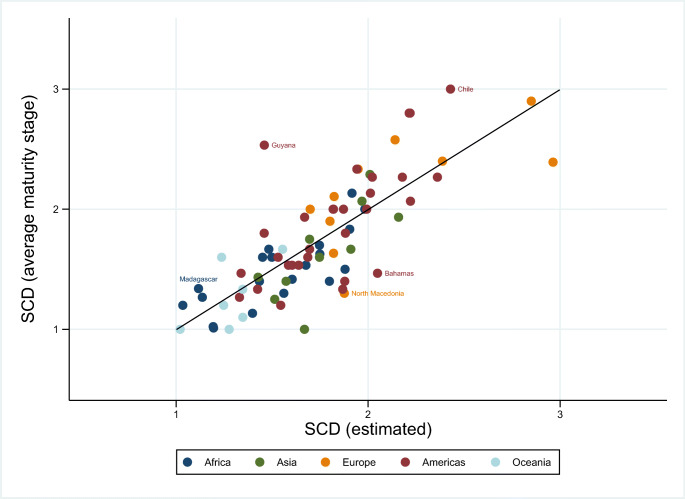
Average maturity stage of the Social and Cultural Dimension (SCD) per nation from the collected indicators in the field and from our estimations

Figure 3 displays the average maturity stage of the SCD per country (vertical axis) and their estimated average maturity stage based on equation (1) (horizontal axis). The black line represents the 45 degrees line where these two values match. The scatter plot shows which countries are above and below the estimated maturity stage according to Eq. (1), identifying those countries over-performing (observations above the 45 degrees line) and those countries under-performing (observations below the line).

Sample countries from all the studied regions fall on both sides of the line, meaning that no region, as indicated at the continent level, helps to understand why some countries were more mature in cybersecurity culture and society than others. While this reinforces our primary finding, the plot raises questions about why some countries over- or under-shoot their predicted levels of maturity.

We selected some countries in our sample to briefly analyse their national CMM report and understand why their average maturity stage of the Social and Cultural Dimension might fall below or above the predicted value. To do so, we considered countries with CMM reports published at the time this paper was written, and whose predicted value was relatively more divergent from the average maturity stage. With these conditions in mind, the chosen countries were Chile, Guyana, The Bahamas (the reports of these three countries are in [25]), Madagascar [19], and North Macedonia [20].

According to the recent data from [25], Chile is a case of an over-performing nation that is on the top distribution of the Social and Cultural Dimension. Given the scale and development levels of Chile when the CMM was implemented, the estimated value was around 2.50 while the maturity stage for this country reached the value 3.00 in all the social and cultural aspects of cybersecurity. When looking at its national profile, [25] emphasizes a rise in concern over cybersecurity among the main social actors in Chile, which brought about collaborative initiatives between the private sector and the government. This served to reinforce the national digital agenda and the provision of e-government services.

We looked at two additional cases of nations over-performing their estimated values for the SCD. The most noticeable case is Guyana, because the indicators of Development and Scale were relatively low for this country when the CMM was implemented. For example, its population had around 783,000 inhabitants, the percentage of Internet users was low (only 37% of its population), and it was classified as an upper-medium income country [40], driving a low estimated score for the SCD. However, even though the country had a shortage of private-sector cybersecurity service providers, the government included ICT as a key component of its Green State Development Strategy to improve government services and business activity [25]. Consequently, the cultural and social aspects were associated with a higher maturity level in Guyana than the predicted by our model, which included the mindset of the government, provision of e-government services, the user understanding of personal information protection online, and the availability of reporting mechanisms.

The other case is Madagascar that, when the CMM was applied in 2016, had a Development value relatively low. Its control of corruption and regulatory quality were rated as relatively low, its income was low [40], and had less than 5% of the population using the Internet. As a result, the estimated maturity stage was close to one. However, the national report shows that some aspects of the SCD had a maturity stage above 1, owing to such initiatives as the emerging availability of e-government services and the establishment of a new legal framework to protect personal data [19].

With respect to observations below the 45 degree line in Fig, 3, The Bahamas had an estimated value for SCD slightly above 2.00 mainly because of its level of Development. For example, 85% of its population are Internet users and its income level is considered high [40]. However, its average maturity stage did not reach the value 1.50, indicating that many of its aspects were in the initial stages of maturity. The information in [25] describes a potential lack of the prioritisation of cybersecurity in the public sector. For example, although e-government services had been part of their digital agenda since 2003, users’ trust and confidence in such services was judged to be low in the report. Also, their National Security Strategy (NCS) began to be developed in 2014, but the strategy was still not yet in place at the time of the report. The relatively low rating of regulatory quality (value −0.05) reinforces this observation.

The case of North Macedonia is similar as its estimated value for SCD was relatively high (1.87) given its Scale and Development variables when the CMM was implemented in 2018. For example, 79 percent of the nation’s population were Internet users and although the control of corruption was not high, the government was perceived to have a good ability to formulate and implement policies and regulations. Despite this relatively high prediction, only a minority of social and cultural aspects of cybersecurity did not have the minimum maturity level, such as in respect to the availability of reporting mechanisms and use of media and social media to inform on cybersecurity. The report described a nation where cybersecurity was not a priority across the main actors of the society given what was then a low level of awareness of cybersecurity risks.

Overall, these six cases show nations under and over-performing in the Social and Cultural Dimension (SCD) with different national approaches to cybersecurity. We examined two cases of over-performing nations on SCD without a NCS in place when they had the CMM review (Guyana and Madagascar). The national CERT of Chile and Guyana were part of CSIRT Americas when the CMM was implemented [25]. The CMM report of North Macedonia reflected the national commitment to implement in the near future the obligations of the European Union Directive on Security and Information Systems in its national legislation [20]. We therefore found the relationship between the maturity of the SCD and the maturity stage in other cybersecurity dimensions to be informative of successes and challenges in each nation, suggesting the potential value of a deeper analysis for feedback to the respective nations.

## Summary and conclusions

This study sought to understand the social and cultural underpinnings of enhancing cybersecurity capacity, and the particular role that regions might play in shaping the maturity of this dimension of capacity building.

The Cybersecurity Capacity Maturity Model for Nations (CMM) identifies attitudes, values, and practices of Internet users in households, business and industry, and government that are directly related to cybersecurity. Based on field research data in 78 nations reviewed under the CMM framework, we observe that our sample countries in Europe and Latin American and the Caribbean were, in average, more mature on these cultural and social aspects of cybersecurity than the countries in our sample within other regions. In line with this observation, these two regions are very active in cybersecurity initiatives within their respective regions. For example, the CSIRT Americas platform is a collaborative network for member states of the Organisation of the American States (OAS), and the European Union (EU)’s Directive on security Network and Information Systems is a directive on cybersecurity followed not only by EU member states, but also by other countries in the region aiming to join the EU.^10^

However, while our data shows that these regional differences exist, they appear to be explained once we control for variables measuring the scale of countries and their development (indicated by their economic, democratic, and governmental context). The regional differences become statistically insignificant when controlling for scale and development. However, the analysis led to a new set of questions based on explaining why particular nations under- or over-perform in relation to what would be expected on the basis of their development and scale, and how the different dimensions of cybersecurity can help to explain these levels of over- or under-performance.

We found that social and cultural attitudes among Internet users in less-developed nations lag behind those more developed nations on nearly every element on which we gathered data. The findings of this study therefore emphasize the impact of national development in contrast to any social and cultural breaking effect that might be anchored in regions of the world. And this reinforces earlier research that found very similar values, attitudes, and practices among Internet users across the world—what might be likened to a cybersecurity culture [11].

However, it also suggests that placing more focus on the social and cultural dimensions of cybersecurity in low-income nations might be a cost-effective approach to advancing capacity building. This finding has implications for policy and practice focused on cybersecurity capacity building. For example, greater efforts on building awareness and strong social and cultural perspectives on the Internet could be relatively effective, particularly aimed at those without experience online. Such initiatives could be less costly than more technically led initiatives [10, 14].

Earlier analyses have shown that the wealth of a nation and its Internet infrastructure are two important components shaping the cybersecurity capacity building of countries [8, 9, 14]. Cross-national research has not demonstrated a regional impact on cybersecurity capacity building, but a nation’s overall level of capacity might be so dependent on technological underpinnings, such as the centrality of Internet use, that nationally or regionally rooted social and cultural implications are too marginal to emerge from cross-national analyses.

### Limitations of the study

Although our findings suggest that continents considered as regions do not have an impact when controlling by the development and scale level of countries, these results are limited by the countries and period of time of our dataset. Our sample of 78 nations is limited in not being a random or comprehensive sample of all countries composing the continents. Moreover, there may be other regional effects we did not observe, such as in a more reduced territory (within industries, organisations, or between the councils of neighbour municipalities, for example). We are limited by our data at a national level that does not allow us to explore the capacity maturity at a less aggregate level.

It has been argued by many, such as Thomas Hylland Eriksen [16], that nations do not represent the most appropriate level of analysis for many social and cultural aspects of a society. Some of our factors would ideally be observed at the individual/human factor level [23], such as a cybersecurity mindset or trust on the Internet. That said, aligning surveys with the national reviews has not yet been financially viable and we have found that experts in cybersecurity have been able to make broad generalizations about Internet users in nations on such issues. If Internet users are broadly judged to lack an awareness of cybersecurity risks, this is important to gauge, even though there is no doubt variation across individuals. Moreover, if we moved our analysis to subunits, similar problems exist, such as studies of capacity building in US states [37]. Moreover, critical policy decisions are being made by national governments. In order to be considered by national policy makers and practitioners, it is valuable to have a national and comparative perspective.

Finally, the methodology to gauge the maturity stage of nations can be another limitation as it relies on judgemental ratings, such as we illicit through the modified-focus groups. The information collected to estimate the national maturity stage of each cybersecurity aspect depends on how collaborative the national participants are during consultations, the information available and public during the desk research, and the different interpretation of some cybersecurity indicators across participants. However, the CMM offers a general framework that allows cross-nationally equivalent reviews to be conducted in countries with very different contexts such that they can be compared overtime and cross-nationally. It balances the trade-offs of using a national level of analysis by using a methodology based on evidence, documentation, and the inclusion of the views of as many stakeholder groups as possible in order to triangulate information from consultations and mitigate any biased conclusions.

### Directions for research

The GCSCC is in a constant state of improving and evolving its CMM and field research methodologies, such as in efforts to provide more objective indicators, less tied to more general judgemental ratings. That said, the results of comparative analyses support the value of the model and methods, given the face validity of the findings, and the transparency of the CMM and its deployment. This study of the social and cultural dimension of capacity building will lead us to focus more attention on under- and over-performing nations on each of the dimensions of the CMM.

## Data Availability

Due to the nature of the project, a subset of governments agreed to keep their data confidential and do not release it. Therefore, we cannot share the dataset due to ethical and legal restrictions. Although we disclose the names of some countries in the submitted manuscript, this information is confidential for a subset of these nations and we would need approval from representatives of the respective nations before publishing such information.

## Footnotes

1 There is more information on the EU Cybersecurity Strategy at https://digital-strategy.ec.europa.eu/en/policies/cybersecurity-strategy, and more information on the OAS cybersecurity initiatives at https://www.oas.org/en/topics/cyber_security.asp.

2 Given the increasing demand for CMM reviews, some country reviews have been implemented by strategic, implementation, or regional partners of the GCSCC (the World Bank, the International Telecommunication Union, the Commonwealth Telecommunications Organisation, NRD Cyber Security, Oceania Cyber Security Centre, and Cybersecurity Capacity Centre for Southern Africa) following the same methodology, including modified-focus groups and interviews to national stakeholders.

3 During the sessions, the Chatham House Rule was used to promote the openness of a discussion, leading to the non-attribution of specific statements. https://www.chathamhouse.org/chatham-house-rule, accessed on April 28, 2020.

4 In June 2020, there was a national CMM review that adjusted the primary field research approach to the conditions of the COVID-19 pandemic. Given restrictions on travelling and social distancing at that time, the interviews and focus-group consultations were undertaken online.

5 Some factors in the CMM dimension Cybersecurity Culture and Society have only one aspect (2.2 Cybersecurity Awareness, 2.5 User Understanding of Personal Information Protection, 2.6 Reporting Mechanism, and 2.7 Media and Social Media), resulting in these factors having the same value as their unique aspects. Table 3 and other tables and figures in this paper avoid duplication of information by providing only the factor’s maturity stage.

6 Secondary data on GDP per capita, total population, and percentage of Internet users from [41]. We calculated the number of users based on the percentage of users and the total population for each nation. Data available until 2019; we used the most recent value available for missing years. For Kosovo, we obtained the percentage of Internet users from [36].

7 This dataset counts the number of years of democracy that at least a country has from 1800 to 2007 [4]. We took the updated version of this dataset considering the extended period until 2015, available at ourworldindata.org and https://sites.google.com/site/mkmtwo/data. In case a democratic country was reviewed under the CMM after 2015, we added the corresponding additional years of democracy.

8 The data analysed in [28] is available in [42] until 2019; we used the most recent value available for missing years.

9 A more detailed level of analysis of the aspects of the CMM dimension on cybersecurity culture and society, not reported here, also show Europe is relatively high on all the specific aspects of cultural and social maturity and Oceania relatively low, with few exceptions.

10 You can find more information on the cybersecurity initiatives in the EU at https://ec.europa.eu/digital-single-market/en/cyber-security, and on OAS initiatives at https://www.oas.org/en/topics/cyber_security.asp.
